# Innate antiviral immunity is impaired in young patients with hand foot and mouth diseases

**DOI:** 10.1186/1753-6561-5-S6-P45

**Published:** 2011-06-29

**Authors:** Y Yang

**Affiliations:** 1 Children’s Hospital of Fudan University, Shanghai, China

## Introduction / objectives

This study was designed to explore the expressions of three pattern-recognition receptors (Toll-like receptor 3, retinoic acid inducible gene-I and melanoma differentiation-associated gene 5) and components of their signaling pathways in the peripheral blood mononuclear cells of children patient with Hand, foot and mouth disease.

## Methods

98 HFMD patients (aged of 1-5 years) and 55 age-matched non-infection children were enrolled in this study; the patients were divided into two groups according to clinical characteristics - with or without complications. The expressions of TLR3, RIG-I, MDA5, IRF-1 and IFN-alpha mRNA were detected by Real-Time PCR.

## Results

The expression levels of TLR3 mRNA in HFMD patients were significantly reduced (6.05±1.26) compared with the non-infection children (7.05±0.96), P<0.001, and the furthermore decreased was found in the patients with complications (5.79±1.15). While, the expressions of MDA5 mRNA in all patients including without complications (4.64±0.49) and with complications (4.60±0.48) were markedly higher than the non-infection children (4.16±0.35), P<0.001. However, RIG-I mRNA was detected only in 72/98 patients, which was not found in the non-infection children. IFN-alpha was lower in the patients without complications (5.71±1.26) than the non-infection children (6.19±0.86), and significantly decreased IFN-alpha mRNA transcriptions were found in the patients with complications (5.54±1.18), compared with the non-infection children P<0.05. Moreover, the changes of IRF-1 mRNA were similar with IFN-alpha, an evidently reduced level of IRF-1 was in the patient with complications (4.89±0.66) compared with the non-infection children (5.32±0.64), P=0.001.

## Conclusion

It is suggested that innate antiviral immunity is impaired in patients and is possibly correlated with the severity of illness.

## Disclosure of interest

None declared.

